# Supplementary material for: Raising Epidemiological Awareness: Assessment of Measles/MMR Susceptibility in Highly Vaccinated Clusters within the Hungarian and Croatian Population—A Sero-Surveillance Analysis
Source: Vaccines (Basel). 2024 May 1;12(5):486. doi: 10.3390/vaccines12050486 (PMC11125914; doi:10.3390/vaccines12050486)
Supplement: Supplementary file 1 [file vaccines-12-00486-s001.zip › vaccines-2980952-supplementary.pdf]

Supplementary Table S1. Schematic representation of the fundamental workflow of the MMR ELISA assay protocol.

Antibodies to be detected	ANTI- MEASLES	ANTI- MUMPS	ANTI- RUBELLA
**Coating antigens, concentrations**	Bio-Rad PIP013 Measles virus, Edmonston strain: 2.8 μg/mL	Bio-Rad PIP014 Mumps virus, Enders strain: 3 μg/mL	Bio-Rad PIP044 Rubella virus, HPV-77 strain: 0.4 μg/mL
**Biomolecule immobilization**	ELISA 96-well Maxisorp plates (Nunc) were coated overnight at 4-6 °C with 100 μL/well. Antigens are dissolved in ELISA Coating Buffer (Bio-Rad BUF030).		
**Saturation of non-specific binding sites**	Blocking: ≥ 2 hours, room temperature, using a combination of non-ionic detergents and proteins in 1:2 ratios (e.g. bovine skin gelatine : purely synthetic, PVA-based blocking buffer)		
**Sample pre-analytics**	Thawing and subsequent preliminary dilution of samples (at a ratio of 1:50) employing IgM Reducing Assay Diluent (Bio-Rad BUF038), aim to minimize background interference and aid in achieving consistent matrix conditions in challenging serum and plasma samples. Following a 15-minute incubation period (room temperature) and subsequent centrifugation, the resulting supernatant is transferred to microplates, achieving a final dilution of 1:200, utilizing washing buffer as the diluent.		
**Standard /quality control reagent (S1-S5)**	3rd WHO International Standard for Anti-Measles (NIBSC code: 97/648)	Anti-Mumps Quality Control Reagent Sample 1 (NIBSC code: 15/B664)	Anti-Rubella Immunoglobulin 1st WHO International Standard Human (NIBSC code: RUBI-1-94)
**Additional in-house controls applied**	Negative Control (NC): sample tested negative in a previous run, Positive Control (PC): sample tested positive in a previous run, blank (sample diluent)		
**Incubation (for primary, secondary antibodies and substrate solution)**	3 x 17 minutes, 37°C		
**Colour detection**	Polyclonal anti-human IgG HRP-conjugated (Dako polyclonal rabbit anti-human IgG or equivalent) + TMB		
**Additional reagents**	TWEEN® 20 (also used as temporary blocker and an integral component of the washing solution) and PBS containing Washing Buffer (WB)		
**Automation and reading**	Siemens BEP 2000 Advance System, λ = 450/620 nm		

Supplementary Table S2. Analysis of statistically significant differences (non-overlapping confidence intervals, CI 95%) among adjacent and non-adjacent age cohorts in Hungary and Croatia.

Base of comparison	Non-overlapping confidence intervals between age groups		
Measles, Hungary, adjacent age groups	20-30/30-40		
	40-50/50-60		
Measles, Hungary, non-adjacent age groups	10-20/>70		
	20-30/50-60	20-30/>70	
	30-40/50-60	30-40/60-70	30-40/>70
	40-50/60-70	40-50/>70	
Measles, Croatia, adjacent age groups	10-20/20-30		
Measles, Croatia, non-adjacent age groups	10-20/40-50		
	20-30/50-60	20-30/60-70	20-30/>70
	30-40/50-60	30-40/60-70	30-40/>70
	40-50/60-70	40-50/>70	
Mumps, Hungary, adjacent age groups		N.A.	
Mumps, Hungary, non-adjacent age groups	20-30/50-60	20-30/60-70	
	30-40/50-60	30-40/60-70	
Mumps, Croatia, adjacent age groups		N.A.	
Mumps, Croatia, non-adjacent age groups		N.A.	
Rubella, Hungary, adjacent age groups		N.A.	
Rubella, Hungary, non-adjacent age groups		N.A.	
Rubella, Croatia, adjacent age groups	40-50/50-60		
Rubella, Croatia, non-adjacent age groups	30-40/50-60		

Supplementary Table S3. Clopper-Pearson exact binomial confidence intervals were computed as a statistical method. The absence of overlap in confidence intervals (CI 95%) was interpreted as indicative of a statistically significant difference between the respective age groups.

Age	N	Non-Event	Event	Event Rate	Lower Limit	Upper Limit	Country	Type
10-20 years	682	52	630	0.924	0.901	0.943	HU	Measles
20-30 years	517	54	463	0.896	0.866	0.921	HU	Measles
30-40 years	313	60	253	0.808	0.760	0.850	HU	Measles
40-50 years	383	49	334	0.872	0.834	0.904	HU	Measles
50-60 years	248	7	241	0.972	0.943	0.989	HU	Measles
60-70 years	257	14	243	0.946	0.910	0.970	HU	Measles
≥ 70 years	280	7	273	0.975	0.949	0.990	HU	Measles
10-20 years	143	17	126	0.881	0.817	0.929	CRO	Measles
20-30 years	279	68	211	0.756	0.702	0.806	CRO	Measles
30-40 years	359	81	278	0.774	0.728	0.817	CRO	Measles
40-50 years	307	82	225	0.733	0.680	0.782	CRO	Measles
50-60 years	291	34	257	0.883	0.841	0.918	CRO	Measles
60-70 years	253	17	236	0.933	0.895	0.960	CRO	Measles
≥ 70 years	132	10	122	0.924	0.865	0.963	CRO	Measles
10-20 years	220	17	203	0.923	0.879	0.954	HU	Mumps
20-30 years	266	34	232	0.872	0.826	0.910	HU	Mumps
30-40 years	159	29	130	0.818	0.749	0.874	HU	Mumps
40-50 years	205	21	184	0.898	0.848	0.936	HU	Mumps
50-60 years	116	4	112	0.966	0.914	0.991	HU	Mumps
60-70 years	122	4	118	0.967	0.918	0.991	HU	Mumps
≥ 70 years	111	8	103	0.928	0.863	0.968	HU	Mumps
10-20 years	143	20	123	0.860	0.792	0.912	CRO	Mumps
20-30 years	279	44	235	0.842	0.794	0.883	CRO	Mumps
30-40 years	359	74	285	0.794	0.748	0.835	CRO	Mumps
40-50 years	307	69	238	0.775	0.724	0.821	CRO	Mumps
50-60 years	291	43	248	0.852	0.806	0.891	CRO	Mumps
60-70 years	253	39	214	0.846	0.795	0.888	CRO	Mumps
≥ 70 years	132	19	113	0.856	0.784	0.911	CRO	Mumps
10-20 years	220	15	205	0.932	0.890	0.961	HU	Rubella
20-30 years	266	30	236	0.887	0.843	0.923	HU	Rubella
30-40 years	159	18	141	0.887	0.827	0.932	HU	Rubella
40-50 years	205	22	183	0.893	0.842	0.932	HU	Rubella
50-60 years	116	11	105	0.905	0.837	0.952	HU	Rubella

60-70 years	122	5	117	0.959	0.907	0.987	HU	Rubella
≥ 70 years	111	4	107	0.964	0.910	0.990	HU	Rubella
10-20 years	143	11	132	0.923	0.867	0.961	CRO	Rubella
20-30 years	279	37	242	0.867	0.822	0.905	CRO	Rubella
30-40 years	359	60	299	0.833	0.790	0.870	CRO	Rubella
40-50 years	307	42	265	0.863	0.820	0.900	CRO	Rubella
50-60 years	291	18	273	0.938	0.904	0.963	CRO	Rubella
60-70 years	253	27	226	0.893	0.849	0.929	CRO	Rubella
≥ 70 years	132	17	115	0.871	0.802	0.923	CRO	Rubella
